# Apatinib as maintenance therapy in extensive-stage small-cell lung cancer: results from a single-center retrospective study

**DOI:** 10.1007/s00432-018-2764-8

**Published:** 2018-10-06

**Authors:** Xiangtao Yan, Qiming Wang, Huijuan Wang, Peng Li, Guowei Zhang, Mina Zhang, Xuanxuan Zheng, Jinpo Yang, Xiaojuan Zhang, Zhiyong Ma

**Affiliations:** Department of Internal Medicine, Henan Cancer Hospital, The Affiliated Cancer Hospital of Zhengzhou University, Zhengzhou, 450008 China

**Keywords:** Small-cell lung cancer, Apatinib, VEGFR-2 tyrosine kinase inhibitor, Maintenance therapy

## Abstract

**Purpose:**

To evaluate the efficacy of maintenance apatinib after chemotherapy for extensive-stage (ED) small-cell lung cancer (SCLC).

**Patients and methods:**

This was a retrospective analysis of 23 cases of extensive-stage SCLC admitted to the Affiliated Cancer Hospital of Zhengzhou University from January 2015 to December 2017. The patients without progression after induction chemotherapy received apatinib 250 mg per day until disease progression or unacceptable toxicity occurred. We analyzed the median progression-free survival (PFS), median overall survival (OS) and safety.

**Results:**

Of 23 enrolled patients, 1 was lost to follow-up. The median PFS from the time of maintenance therapy was 4.1 months (95% CI 3.63–4.57 months). The median PFS from the time of induction chemotherapy was 8.3 months (95% CI 7.20–9.40 months). The median OS from the time of maintenance therapy was 12.5 months (95% CI 5.51–19.49 months). The median OS from the time of induction chemotherapy was 17.0 months (95% CI 9.86–24.14 months). The most frequent treatment-related adverse events were hand–foot syndrome (43.5%, 10/23) and secondary hypertension (30.4%, 7/23), followed by fatigue, proteinuria, nausea, and oral mucositis (17.4%, 13.0%, 13.0%, and 8.7%, respectively). Hematologic toxicity included thrombocytopenia (30.4%), leucopenia (26.1%), and anemia (17.4%). The main grade 3 or 4 toxicities were hand–foot syndrome (8.7%, 2/23) and hypertension (4.3%, 1/23).

**Conclusion:**

Maintenance apatinib was safe and achieved encouraging PFS and OS in extensive-stage SCLC.

## Introduction

Small-cell lung cancer (SCLC) accounts for approximately 10–15% of the total number of lung cancer cases and has a poor outcome (van Meerbeeck et al. 2011). In extensive-stage (ED) small-cell lung cancer, the 5-year survival rate is less than 5% (Albain et al. 1991). The main cause of treatment failure is rapid recurrence and resistance to second-line chemotherapy (Schiller et al. 2001), although we can achieve a very high response rate with first-line chemotherapy. The progression-free survival (PFS) and overall survival (OS) are very poor in extensive-stage small-cell lung cancer (Demedts et al. 2010; Noda et al. 2002). To improve the survival outcome, a number of therapeutic approaches have been tried, such as dose dense chemotherapy (Masutani et al. 2000), high-dose chemotherapy (Leyvraz et al. 1999), maintenance therapy, and consolidation chemotherapy (Rossi et al. 2010; Yang et al. 2016). However, the results showed no proven benefit. Therefore, there is still an urgent need for the evaluation of novel agents to improve disease outcomes.

Previously, Lucchi et al. reported that high microvessel density and vascular endothelial growth factor (VEGF) protein expression correlated with poor clinical outcome in patients with limited stage SCLC (Lucchi et al. 2002). Patients with lower pretreatment circulating VEGF levels were more likely to respond to chemotherapy compared to those with higher levels of VEGF (Salven et al. 1998). The results from these studies suggest that VEGF may be linked to overall poor outcome in SCLC. Therefore, the inhibition of VEGF represents a rational therapeutic strategy for evaluation in SCLC. Moreover, these drugs have generally been considered ideal for the maintenance approach, because they are well tolerated and conveniently administered for long-term use. However, a phase II trial of sorafenib in conjunction with chemotherapy as maintenance therapy in extensive-stage small-cell lung cancer showed that combination therapy has significant toxicity at current dose levels and is associated with disappointing efficacy data (Sharma et al. 2014).

Apatinib is a tyrosine kinase inhibitor that selectively inhibits the vascular endothelial growth factor receptor-2 (VEGFR2). It is an orally bioavailable, small molecule agent that is thought to inhibit angiogenesis in cancer cells. Although there have been few studies of small-cell lung cancer treated with apatinib, a retrospective study showed that apatinib exhibits modest activity and acceptable toxicity for heavily pretreated patients with extensive-stage small-cell lung cancer. The disease control rate was 81.8% (Hong et al. 2017).

However, whether there is a survival benefit with apatinib as maintenance therapy in extensive-stage small-cell lung cancer is unclear. Therefore, the aim of this study was to analyze the efficacy and safety of apatinib as maintenance therapy in extensive-stage small-cell lung cancer.

## Patients and methods

### Clinical data

The clinical data of 23 patients with extensive-stage SCLC without progression (according to response evaluation criteria in solid tumors 1.1) after induction chemotherapy who were admitted to the Affiliated Cancer Hospital of Zhengzhou University from January 2015 to December 2017 were collected. Eligible patients had histologic documentation of SCLC and extensive-stage disease (extrathoracic metastatic disease, malignant pleural effusion, contralateral supraclavicular adenopathy, or contralateral hilar adenopathy) according to the UICC (The Union for International Cancer Control)’s 8th lung cancer TNM classification. The baseline assessment was completed within 1 week before the start of treatment and included thoracic and abdominal imaging examinations and blood routine and biochemical examinations. Eligibility criteria included Eastern Cooperative Oncology Group (ECOG) performance status of 0 to 2 and normal initial laboratory tests. Patients with active brain metastases, carcinomatous meningitis, and spinal cord compression were excluded (patients with brain metastases could be enrolled if they had no symptoms, did not need dehydration and glucocorticoid treatments, and did not need radiotherapy in the short term. In addition, patients with brain metastases who completed the treatment 21 days before medication and had stable symptoms could be enrolled as well).

There were 15 males and 8 females with the median age of 60 years (with the range of 47–71 years). There were 16 cases with an ECOG PS score of 0–1 and 7 cases with an ECOG PS score of 2. There were seven cases combined with hepatic metastases and eight cases combined with brain metastases. There were 8 cases and 15 cases with TNM stage IVa and stage IVb disease, respectively, of which 16 cases later underwent second-line chemotherapy. The baseline characteristics are shown in Table 1.

**Table 1:** Baseline data of patients

Characteristics	No. (%)
Patients enrolles	23
Sex (male/female)	15/8 (65.2/34.8)
Median age (years)	60
Range	47–71
Smoking history	11 (47.8)
ECOG PS	
0–1	16 (69.6)
2	7 (30.4)
Hepatic metastases	7 (30.4)
Brain metastases	8 (34.8)
TNM staging	
IVa	8 (34.8)
IVb	15 (65.2)
Second-line chemotherapy	16 (69.6)

### Treatment methods

All cases received 2–6 cycles of induction chemotherapy (cisplatin 75 mg/m^2^ or carboplatin area under the curve of 5 on day 1 plus etoposide 100 mg/m^2^ per day on days 1–3 every 21 days). Then, 250 mg of apatinib was orally administered once daily after the completion of chemotherapy (> 21 days and ≤ 42 days from the first day of the last chemotherapy) for patients with a therapeutic efficacy achieving complete response (CR), partial response (PR), or stable disease (SD). Three cases received less than four cycles of induction chemotherapy because of poor chemotherapy compliance and four cases received less than four cycles, because they did not achieve CR or PR. Concurrent whole brain radiotherapy was conducted for all eight cases with brain metastases at the same time as induction chemotherapy. In the 15 cases without brain metastases, one case underwent prophylactic cranial irradiation. Fewer patients had prophylactic cranial irradiation, thus, they were not included in the statistical analysis. After disease progression, 16 cases underwent second-line treatment.

### Observation indicators

Physical examination was performed every 3 weeks, including blood routine and biochemical examinations as well as electrocardiograms. Imaging assessments, such as doppler ultrasound, CT, or MRI, were conducted every 6 weeks.

### Adverse events

Adverse events were evaluated based on the evaluation standards of common adverse reaction events in NCI-CTCAE 4.0, which were divided into grades I–V. In this study, grades III–IV were defined as moderate adverse events, and grade V was defined as death.

### Survival

Progression-free survival (PFS) and overall survival (OS) were determined from the time of induction chemotherapy and from the time of maintenance therapy. PFS from the time of induction chemotherapy (maintenance therapy) was defined as: the time from the start of induction chemotherapy (maintenance therapy) to the time of disease progression or death by follow-up. Moreover, patients lost to follow-up or patients without disease progression were treated according to censored data, and the time of censoring was the last follow-up time to confirm that there was no disease progression. OS from the time of induction chemotherapy (maintenance therapy) was defined as: the time from the start of induction chemotherapy (maintenance therapy) to the time of death by follow-up. Moreover, patients lost to follow-up and patients who survived were treated based on censored data, and the time of censoring was the last follow-up time to confirm death.

### Statistical analysis

Statistical analysis was performed using SPSS17.0 statistical software. Progression-free survival and overall survival were analyzed using Kaplan–Meier survival curves. Multivariate survival analysis used a multivariate cox regression model. *α* = 0.05 was used as the level of significance in all statistical analyses.

## Results

### PFS

**Fig. 1:**
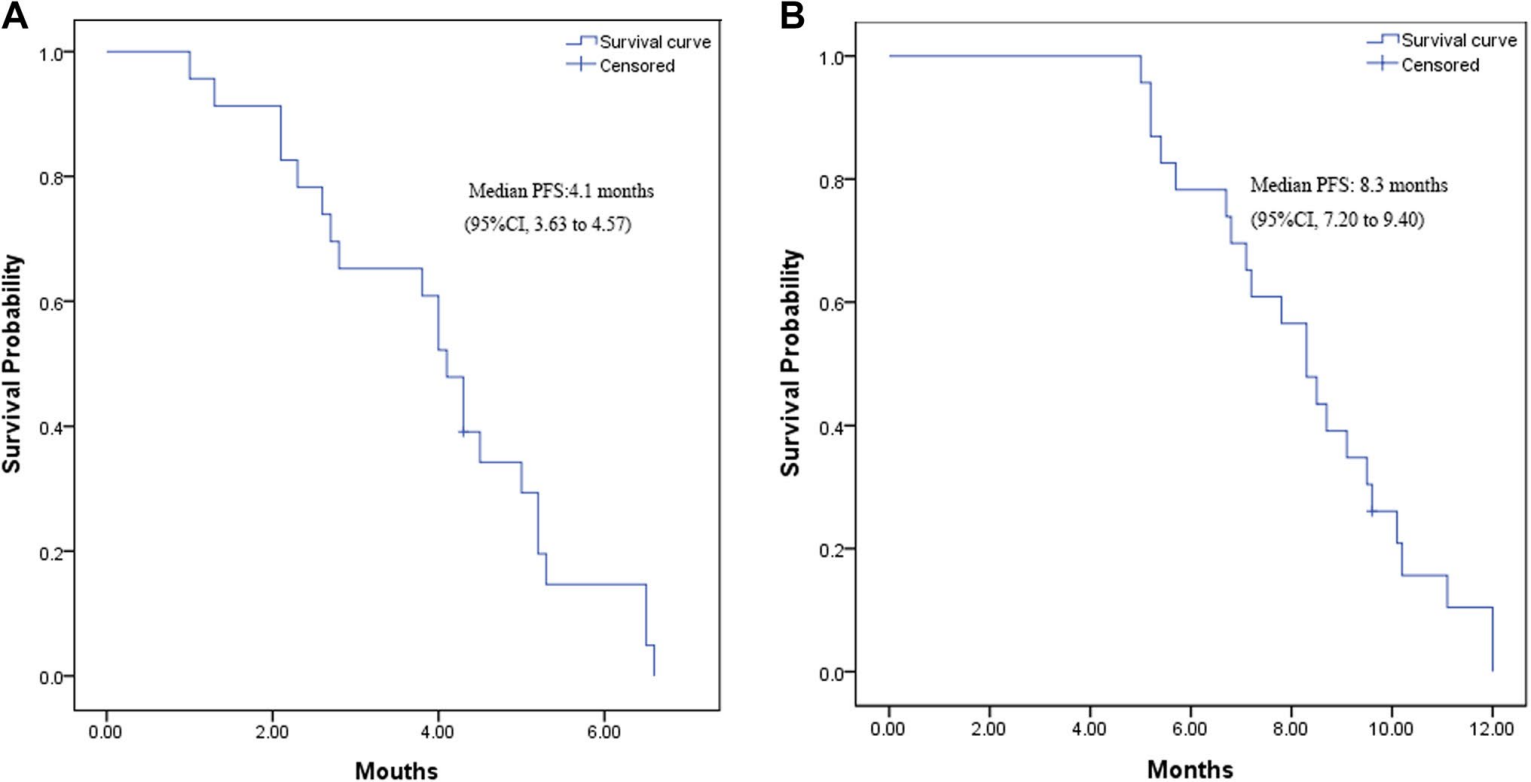
**a** Kaplan–Meier curve for progression-free survival from the time of maintenance therapy. **b** Kaplan–Meier curve for progression-free survival from the time of induction chemotherapy

Of 23 enrolled patients, 1 was lost to follow-up. The median PFS from the time of maintenance therapy was 4.1 months (95% CI 3.63–4.57 months; Fig. 1a). The median PFS from the time of induction chemotherapy was 8.3 months (95% CI 7.20–9.40 months; Fig. 1b).

### OS

**Fig. 2:**
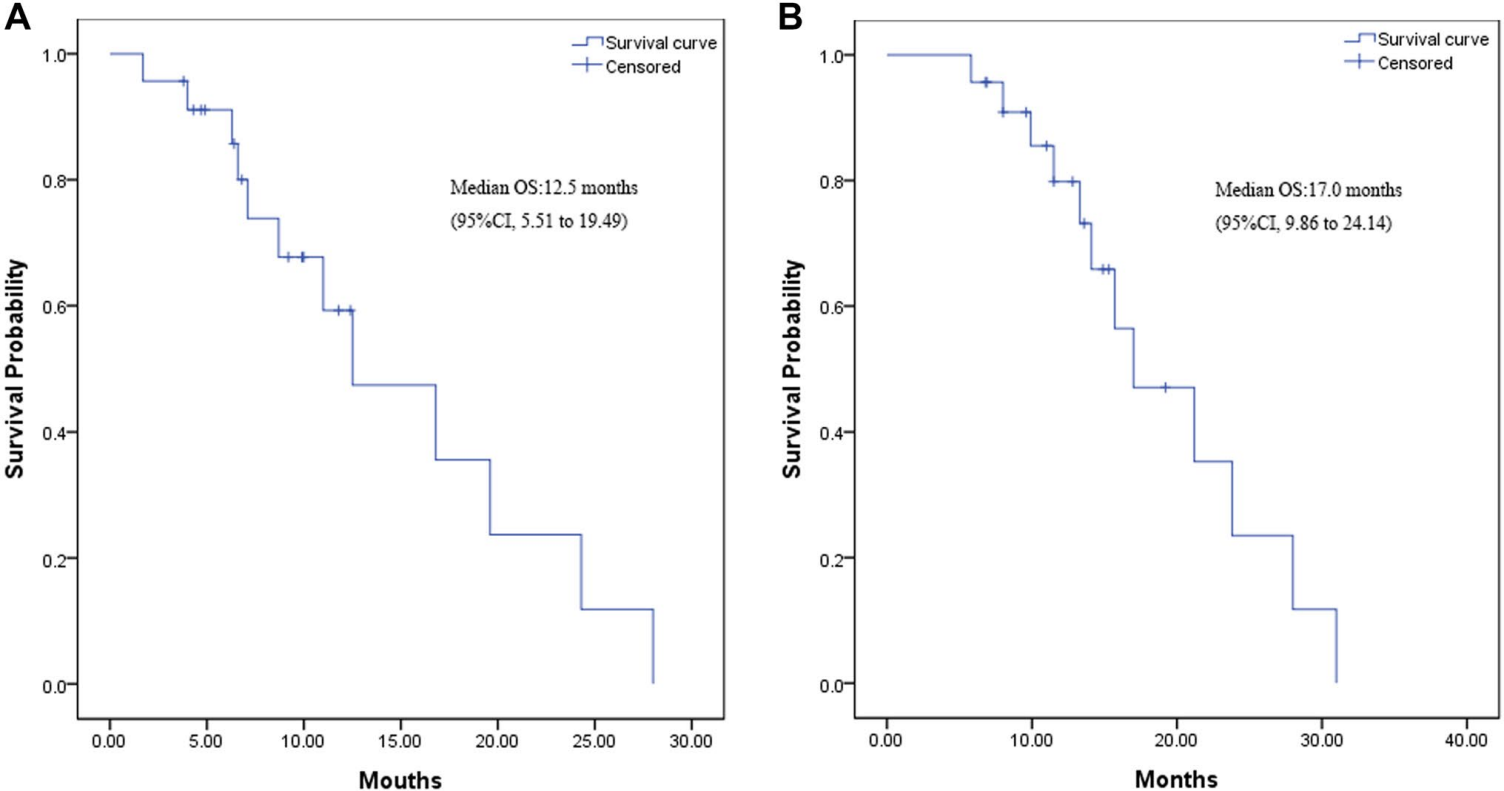
**a** Kaplan–Meier curve for overall survival from the time of maintenance therapy. **b** Kaplan–Meier curve for overall survival from the time of induction chemotherapy

The median OS from the time of maintenance therapy was 12.5 months (95% CI 5.51–19.49 months; Fig. 2a). The median OS from the time of induction chemotherapy was 17.0 months (95% CI 9.86–24.14 months; Fig. 2b).

Multivariate Cox regression analysis showed that brain metastases were significantly correlated with OS in PS score, age, gender, smoking status, hepatic metastases, and brain metastases. The risk of death for patients with brain metastases increased significantly (HR 9.725; 95% CI 1.770–53.444; *P* = 0.009).

### Adverse events

Compared with the previous clinical data, the occurrence of treatment-associated adverse events in this study was more optimistic. The most common adverse event was hand–foot syndrome with an incidence rate of 43.5%, followed by secondary hypertension with an incidence rate of 30.4%. The incidence rates of fatigue, proteinuria, nausea and oral mucositis were 17.4%, 13.0%, 13.0%, and 8.7%, respectively. Regarding hematological adverse events, the most common event was thrombocytopenia with an incidence rate of 30.4%. The incidence rates of leukopenia and anemia were 26.1% and 17.4%, respectively. Grade III/IV adverse events included hypertension and hand–foot syndrome, which had incidence rates of 4.3% and 8.7%, respectively. No other unintended adverse events occurred besides abdominal pain in one patient. Treatment-associated death was not observed (Table 2).

**Table 2:** Summary of adverse events

	Total (%)	Grade I	Grade II	Grade III	Grade IV	Grade III/IV (%)
Secondary hypertension	7 (30.4)	3	3	1	0	1 (4.3)
Proteinuria	3 (13.0)	2	1	0	0	0
Hand–foot syndrome	10 (43.5)	8	0	2	0	2 (8.7)
Oral mucositis	2 (8.7)	1	1	0	0	0
Fatigue	4 (17.4)	2	2	0	0	0
Nausea	3 (13.0)	2	1	0	0	0
Bellyache	1 (4.3)	0	1	0	0	0
Thrombocytopenia	7 (30.4)	5	2	0	0	0
Leucopenia	6 (26.1)	6	0	0	0	0
Anemia	4 (17.4)	3	1	0	0	0

## Discussion

Although many experiments have been performed, there has been no breakthrough regarding the overall survival of patients with small-cell lung cancer. The median survival of extensive-stage small-cell lung cancer remains at approximately 7–12 months (Kalemkerian et al. 2013). One of the most important reasons may be because small-cell lung cancer showed strong drug resistance if progression occurred after first-line treatment, leading to extremely poor efficacy of second-line treatment. Because of the low toxicity and easy administration, targeted therapeutic agents are the best candidates for maintenance therapy. In this study, maintenance therapy was performed using apatinib for patients with extensive-stage small-cell lung cancer who did not suffer from disease progression after first-line treatment. The results showed that the median PFS from the time of maintenance therapy was 4.1 months. The median PFS from the time of induction chemotherapy was 8.3 months. The median OS from the time of maintenance therapy was 12.5 months. The median OS from the time of induction chemotherapy was 17.0 months. In this study, a single-arm cohort analysis was applied, because there were fewer cases. However, compared to the previous data of 7–12 months, the results of this study were significantly advantageous. Clinical results of another study using sunitinib to treat extensive-stage small-cell lung cancer in contrast with maintenance therapy using placebo showed that the median PFS in the sunitinib maintenance therapy group was 3.7 months, while it was 2.1 months in the placebo group (HR 1.62; 95% CI 1.02–2.60; one-side *P* = 0.02). The median overall survival in the sunitinib maintenance therapy group was 9 months, while it was 6.9 months in the placebo group (HR 1.28; 95% CI 0.79–2.10; one-side *P* = 0.16) (Ready et al. 2015). In another randomized phase II–III study of bevacizumab in combination with chemotherapy in previously untreated extensive small-cell lung cancer patients, the results showed that the PFS was 5.3 months and 5.5 months (*P* = 0.82), respectively, in the maintenance group with bevacizumab and the contrast group (Pujol et al. 2015). Horizontal comparison of the study results demonstrated that the data obtained in our study performed well.

In multivariate regression analysis, the only factor that was significantly correlated with survival was brain metastasis. It may be that the cerebrospinal fluid concentration of apatinib was poor. Of course, more evidence is needed. There was no significant association with hepatic metastasis, PS score, age, gender, or staging, suggesting that there was no special selectivity in baseline clinical characteristics using apatinib in the maintenance therapy of patients. Therefore, there was no basis for clinical characteristics in identifying the indications for apatinib maintenance therapy.

Of course, the results of this study had an inevitably large deviation, because the number of cases was small, and this was a retrospective study. However, in view of the good performance of apatinib in the treatment of small-cell lung cancer after second-line treatment, it is necessary to investigate the advantages and disadvantages of maintenance therapy for small-cell lung cancer. Moreover, there is currently no study in this field. This retrospective study was performed to preliminarily investigate the clinical efficacy and safety of apatinib maintenance therapy in extensive-stage small-cell lung cancer, which is of great clinical value. Furthermore, this study provided theoretical and clinical evidence for further prospective and randomized controlled studies.

The appropriate dose of apatinib as a maintenance therapy for small-cell lung cancer is also worthy of further discussion. Apatinib was first approved for use in advanced gastric cancer in China. The recommended dose is 850 mg/day three times per day. With further investigation by clinical and pharmacological scholars, its clinical application has been gradually extended to the treatment of other malignant tumors, including non-small cell lung cancer and small-cell lung cancer. In the stage II clinical experiments before the launch of apatinib that compared patients with other tumors and healthy subjects, drug absorption was delayed and drug exposure was lower for patients with gastric cancer. This may be because the dissolution and absorption of drugs were affected in patients with gastric cancer due to surgical history or overall physical condition (Li et al. 2013). Therefore, the optimal treatment dose of lung cancer cannot refer to that used in gastric cancer. In stage I clinical experiments for apatinib, it was found that doses greater than 250 mg/day would result in hypertension and proteinuria for nearly half of patients (Li et al. 2010). Likewise, in the treatment of extensive-stage small-cell lung cancer after failure of second-line and third-line chemotherapy, 45.5% of patients could not tolerate a dose of 500 mg/day, necessitating reduction of the dose (Hong et al. 2017). In maintenance treatment, controlling toxicity and pursuing efficacy are necessary. Thus, in this study, the maintenance dose was 250 mg/day initially.

The results revealed that the most common adverse events were hand–foot syndrome, secondary hypertension, and thrombocytopenia with incidence rates of 43.5%, 30.4%, and 30.4%, respectively. However, the adverse events of grade III/IV only included hypertension and hand–foot syndrome with incidence rates of 4.3% and 8.7%, respectively. There were no patients who could not tolerate the treatment, and no dose reduction was observed. Thus, clinical tolerance was acceptable at a maintenance dose of 250 mg.

It is well known that targeted drugs act on specific biological targets. However, apatinib is a multi-target inhibitor that mainly acts on VEGFR2. This study lacks corresponding clinical data; thus, we cannot further analyze specific molecular markers. It was mentioned that VEGF protein expression, peripheral blood VEGF level, and the prognosis of patients were associated with a certain extent. Therefore, we can try to add specific molecular markers for analysis in subsequent clinical studies to screen for more effective populations.

The results of this study support the strategy of using novel therapy in the maintenance of extensive-stage SCLC. Apatinib given after the standard chemotherapy in extensive-stage SCLC achieved encouraging PFS and OS. In addition, toxicity control is a very important part of maintenance. Fortunately, the drug was well tolerated, with no serious adverse events. These results support a future multi-center prospective randomized study of maintenance therapy in SCLC.
